# Dance Training and the Neuroplasticity of the Vestibular-Ocular Reflex: Preliminary Findings †

**DOI:** 10.3390/brainsci15040355

**Published:** 2025-03-29

**Authors:** Raghav H. Jha, Erin G. Piker, Miranda Scalzo, Diana Trinidad

**Affiliations:** 1 School of Communication Sciences and Disorders, The University of Memphis, Memphis, TN 38152, USA; 2 Department of Communication Sciences and Disorders, James Madison University, Harrisonburg, VA 22807, USA; pikereg@jmu.edu (E.G.P.); mscalzo98@gmail.com (M.S.); dianatrinidad14@gmail.com (D.T.)

**Keywords:** dancer training, neuroplasticity, vestibulo-ocular reflex, HIMP and SHIMP

## Abstract

Background: The impact of dance training on brainstem-mediated vestibular reflexes remains unclear. This study examined the vestibulo-ocular reflex (VOR) and its suppression during high-speed head movements, which may closely resemble the head-turning speeds used in dancers’ spotting techniques, using the video head impulse test. Methods: Eighteen female college students (mean age: 21 years) were divided into two groups—nine trained dancers (≥six years of dance training) and nine age-matched non-dancers—all without a history of hearing, vestibular, or neurological disorders. VOR function was assessed using the head impulse paradigm (HIMP) and the suppression head impulse paradigm (SHIMP) for right and left lateral stimulation, with minimum head velocities of 150°/s. Results: All participants exhibited VOR measures within normal limits and the VOR gain of dancers did not significantly differ from that of non-dancers. However, most dancers reported a preference for right-sided pirouettes and the right-side SHIMP gain negatively correlated with years of training, suggesting a link between preferred turning direction and VOR suppression ability. Furthermore, dancers with over 15 years of training exhibited earlier anti-compensatory saccade latencies (~75 ms) during SHIMP. Conclusions: Trained dancers maintain a healthy VOR and may develop enhanced voluntary control, enabling more effective VOR suppression. The earlier onset of anti-compensatory saccades suggests neural adaptations in eye–head coordination for high-velocity movements. Given the study’s small sample size and the inclusion of non-fulltime dancers, future research with larger samples of professional dancers is needed for enhanced generalizability. These findings provide preliminary evidence of dance-related neuroplasticity in brainstem-mediated vestibular reflexes and open new research avenues.

## 1. Introduction

Humans have an innate affinity for rhythmic movement, possibly rooted in early experiences such as being rocked in a cradle [1,2]. This predisposition is closely linked to our bipedal locomotion and has contributed to the evolution of dance [1,2]. Throughout history, dance has played a vital role in shaping societal and cultural identities while serving as a medium for information sharing and entertainment [3]. However, dancing is not only an enjoyable activity but also supports systemic health and general well-being. Studies have shown that intensive dance training could induce reorganizational changes in cortical and subcortical structures [4,5]. Dance training has also been associated with improved cognition and postural balance [5,6]. While dance training has been linked to enhanced postural balance, maintaining optimal balance requires an integrated function of proprioception, vision, and the vestibular system. Very few studies have examined how dance training specifically impacts vestibular function, particularly the brainstem-mediated vestibular reflexes such as the vestibular-ocular reflex (VOR). The present study examines the effects of dance training on the VOR as measured with video head impulse testing (vHIT).

The VOR stabilizes vision during head movements by coordinating eye movements. The head motion detected by vestibular end organs is transmitted to the vestibular nuclei, which are then relayed to oculomotor neurons controlling eye muscles. During the VOR, the eyes move at the same velocity as the head but in the opposite direction. Disruptions in the VOR can result in oscillopsia, dizziness, and balance issues, making VOR evaluation an essential part of vestibular diagnostics. A common tool for assessing the VOR is the vHIT, in which a patient’s head is passively moved at a speed of 150–250 degrees per second while they focus on a stationary, Earth-fixed target. VOR function is measured by VOR gain (the ratio of eye velocity to head velocity). Under normal conditions, the VOR gain obtained through the head impulse paradigm (HIMP) is close to 1, indicating the eyes move at the same speed (but in the opposite direction to the head). Reduced VOR gain generally suggests a vestibular lesion.

The ability to suppress VOR is also essential for visual stability, such as when tracking a moving object (e.g., tracking a moving ball while playing tennis). VOR suppression is almost complete at low frequencies (e.g., 0.08 Hz in rotary chair tests), where visual stabilization is mediated by the oculomotor system via saccadic and smooth pursuit movements [7,8]. For higher frequency head movements, VOR suppression is suggested to mediated by a non-visual mechanism [8]. The suppression head impulse paradigm (SHIMP), during vHIT, assesses high-frequency VOR suppression by having a patient maintain their gaze on a head-fixed target during head movement. In healthy individuals, head movement induces an initial VOR response opposite to the head’s direction, followed by an anti-compensatory saccade (in the direction of the head movement) to refocus on the head-fixed target [9]. SHIMP VOR gains are slightly reduced compared to HIMP gains; however, they remain close to 1 [9]. Together, HIMP and SHIMP effectively assess brainstem-mediated VOR function and suppression for high-frequency head movements.

While the HIMP and SHIMP measures are commonly used in vestibular diagnosis, they also serve as measures of adaptive plasticity, often observed in vestibular compensation in patients with unilateral vestibular illness. In acute vestibular disorders, HIMP gain is significantly reduced, with minimal or absent anti-compensatory saccades on SHIMP. As the condition progresses to a chronic stage, both VOR gain and anti-compensatory saccade amplitude steadily increase, indicating reorganization within the vestibular pathways [10]. Vestibular rehabilitation exercises have also been shown to induce reorganizational changes in VOR among patients. Although there is substantial evidence of VOR reorganization in patient populations, it is unclear whether training could produce similar effects in healthy individuals. This raises questions about how long-term training, such as dance training, might impact brainstem-mediated VOR and VOR suppression.

The effects of dance training on vestibular processing are exemplified by ballet dancers’ ability to perform multiple pirouettes with minimal or no sensation of vertigo. Compared to healthy controls, dancers exhibit reduced cortical activity in the cortico-vestibular perception white matter network, which has been linked to their decreased perception of vertigo during pirouettes [11]. Dancers also use spotting, a technique that involves focusing on a fixed point while rotating and quickly realigning the head and eyes with the body. Spotting is commonly practiced in ballet, jazz, and other dance styles and is associated with reduced dizziness during high-velocity turns [12]. In a study of 34 dancers, dizziness perception was significantly lower when spotting was used compared to when it was not [12]. While it is clear that use of the spotting technique reduces the perception of dizziness, the effects of prolonged dance training on the VOR remain unclear. The vHIT is the only vestibular test capable of assessing VOR during high-velocity head movements, such as those involved in spotting. During vHIT, participants’ heads are moved at velocities of 150–200 degrees per second, closely resembling the speed of spotting [13]. We hypothesize that dance training, combined with spotting techniques, may significantly affect VOR at the high frequencies measured by vHIT. We speculate that this may result from dancers’ enhanced cognitive control over sensory-driven motor movements [14], developed through training, which could help them predict and manage eye control during VOR examinations.

A few studies have explored VOR and VOR suppression in dancers [15,16,17]. Osterhammel et al. (1968) [15] observed reduced VOR gain during whole-body rotation in ballet dancers. Teramoto et al. (1994) [16] found greater VOR suppression during whole-body and caloric stimulation, with a correlation between experience and suppression degree. Tanguy et al. (2008) [17] reported a 27% reduction in VOR gain in skaters during rotary chair tests. While these studies highlight differences in VOR gain and suppression between dancers and non-dancers under low- and mid-frequency stimulation, there is limited information on VOR responses to high-frequency head movements. To date, only two studies have examined the effects of dance training on VOR suppression during high-frequency, natural head movements using vHIT [18,19].

Maheu et al. (2019) [18] found no difference in VOR gain between dancers and non-dancers using HIMP but observed significantly higher VOR suppression (reduced SHIMP gain) among dancers at approximately 60 ms post head turn. The authors attributed this finding to potentially enhanced vestibular/cerebellar control resulting from dance training. However, the reduction in SHIMP gain was significant only at 60 ms, with no differences observed at 40 ms or 80 ms; in fact, dancers exhibited a higher mean SHIMP gain at 80 ms. The equipment used in Maheu et al.’s study [18] measured VOR gain as instantaneous gains at 40, 60, and 80 ms, which could be affected by goggle slippage. An alternative method, the area ratio gain, compares the areas of eye and head movement over the full movement trace, reducing errors caused by goggle slippage. In a follow-up study, Moin-Darbari et al. (2023) [19] reported slightly higher VOR HIMP gain in dancers compared to non-dancers using the area ratio method. The authors did not examine SHIMP gain. Given the inconsistencies in HIMP gain results across the two studies using different methods of VOR gain and the differential effects of dance training on SHIMP gain at different time points [18], it would be valuable to replicate the study. The current study aimed to compare VOR gain under the HIMP and SHIMP between dancers and non-dancers, building on Maheu et al. (2019) [18] but employing different equipment and a distinct VOR gain calculation method.

## 2. Methods

### 2.1. Participants

The current study is a revised and expanded version of a conference presentation [20]. The study was approved by the Institutional Review Board (IRB #20-1515) at James Madison University. Participants were recruited from the undergraduate and graduate student populations at the university, and each participant provided written informed consent. A total of 18 female participants, divided into two groups (9 dancers and 9 age-matched healthy non-dancers), were included. None of the participants had a history of ear-related, neurological, or vestibular issues, nor any head trauma or concussion. To qualify as a dancer, participants needed a minimum of six years of dance experience and at least two h of current dance practice per week. Dancers reported the duration of their dance training, their weekly (time in hours) commitment to dance, and preferred turning direction. Dancers were also asked to subjectively rate their confidence in their turning ability on a scale—1 (novice), 2 (intermediate), and 3 (advanced). We asked participants about their dominant-writing hand and found that two dancers and two non-dancers were left-hand dominant. Among the left-hand dominant dancers, one preferred turning to the left and the other to the right, with confidence levels ranging from moderate to advanced, respectively. The mean age of the dancers was 20.88 years (SD = 2.4), and for non-dancers it was 20.00 years (SD = 1.2). A total of 15 individuals with a history of dance training consented to the study, but only 9 met the dancer’s qualification and were included in subsequent analyses.

### 2.2. Dancers Profile

Dancers spent an average of 6.1 (SD = 5.4, range 2–17) h per week practicing dance. Of the nine dancer participants, seven preferred turning to the right, one preferred turning to the left, and one had no directional preference. On a scale of 0–3, most dancers (n = 6) rated their turning confidence as “intermediate” (2) or between “intermediate” and “advanced” (2.5), with an average confidence rating of 2.2 (range: 1–3). Two dancers described their turning confidence to be advanced (3). Dance experience varied from 6 to 18 years, with 8 participants reporting dance training over 10 years.

### 2.3. Procedure

Both the HIMP and SHIMP were conducted on dancers and non-dancers using a Natus ICS Impulse (Middleton, WI, USA) equipment and Otosuite vestibular software version 1.2. Participants sat 1 m from a stationary target mounted on a wall. The eye movement was recorded using a monocular camera mounted on the right eye. Calibration was performed to record precise eye movements. For HIMP testing, participants focused on an Earth-fixed target on the wall while the tester randomly turned their head quickly to the right or left. The minimum head velocity was set at 150 degrees/second, and only head thrusts stimulating the horizontal semicircular canal were accepted; those in other planes or below the minimum velocity were excluded. For SHIMP testing, head thrusts were also conducted with a minimum velocity of 150 degrees/second. For the SHIMP testing, a head-fixed laser target projected from the video goggles served as the focal point, and participants were instructed to maintain focus on the head-fixed target while the head thrusts were administered. A minimum of ten successful trials were obtained for each direction for HIMP and SHIMP.

### 2.4. Statistical Analysis

The Shapiro–Wilk test indicated that most data followed a normal distribution (*p* > 0.05). A two-way mixed ANOVA assessed the effects of group (dancer vs. non-dancer) and condition (right HIMP, left HIMP, right SHIMP, left SHIMP) on VOR gain, followed by post hoc comparisons. Spearman’s correlation tested the relationships between dance experience and HIMP VOR gain and between dance experience and SHIMP VOR gain for both right and left head thrusts.

## 3. Results

### 3.1. VOR Gain Using HIMP and SHIMP

**Figure 1:**
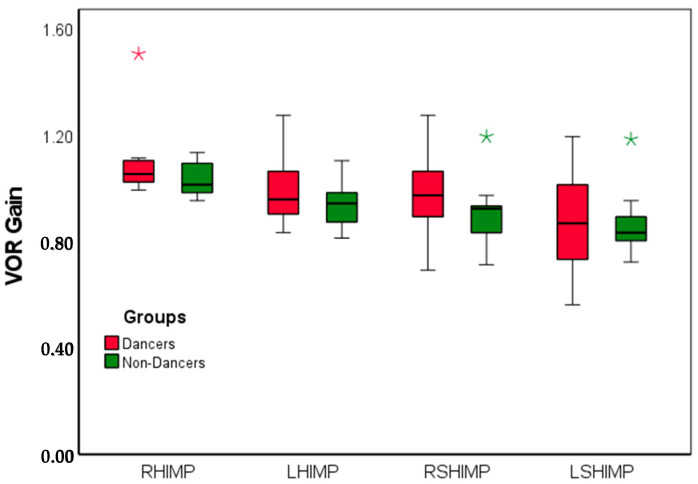
A box plot showing VOR gain for the HIMP as well as the SHIMP for the right and the left ears. RHIMP–right HIMP, LHIMP–left HIMP, RSHIMP–right SHIMP, LSHIMP–left SHIMP. The thick line in the box represents the mean values. The error bars indicate ±2 S.E. The star (*) indicates an outlier.

Both dancers and non-dancers showed healthy VOR gain, (Figure 1), with HIMP gains between 0.8 and 1.2 (Figure 1). On average, the SHIMP gains were lower than the HIMP gains, with dancers exhibiting larger variability on SHIMP gains. A two-way mixed ANOVA was conducted to examine the effect of group (dancer vs. non-dancer) and condition (right HIMP, left HIMP, right SHIMP, left SHIMP) on VOR gain. The results indicate that there was no significant main effect of group (F_(1,16)_ = 0.68, *p* = 0.421), suggesting no overall difference in VOR gain between the two groups. A significant effect of condition was seen on the VOR gain, (F_(3,24)_ = 25.931, *p* = 0.001). Post hoc pairwise comparisons (with Bonferroni correction) indicated that VOR gain was significantly higher in right HIMP compared to left HIMP (*p* = 0.001), right SHIMP (*p* = 0.001), and left SHIMP (*p* < 0.001). No other comparisons reached statistical significance. The interaction effect between group and conditions was not significant, (F_(3,48)_ = 0.28, *p* = 0.84), suggesting that the pattern of VOR gain across conditions did not differ between groups.

### 3.2. Dance Experience as a Continuous Variable

**Figure 2:**
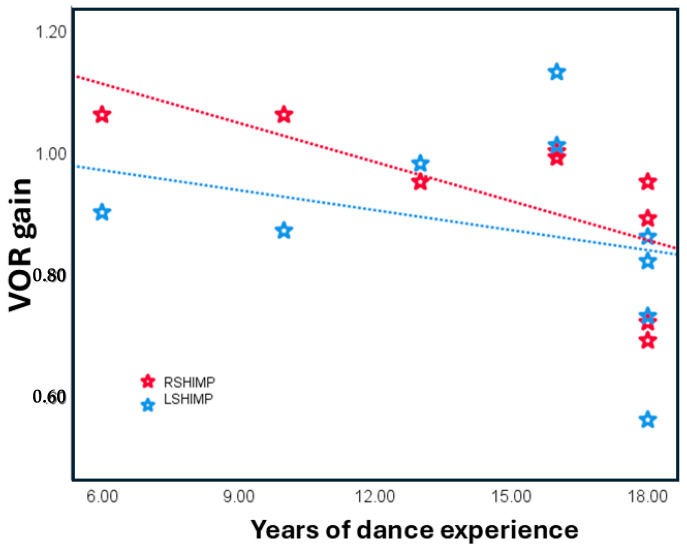
Scatter plot showing left and right SHIMP gain across years of dance training.

Spearman’s correlation in the dancers’ group assessed the relationship between VOR gain and years of dance experience. A significant negative correlation was found between years of dance training and right-side SHIMP gain (r = −0.697, *p* = 0.04), but not for the left side (r = −0.284, *p* = 0.46). No significant correlation was observed between dance experience and HIMP gain on either the right (r = 0.10, *p* = 0.78) or left side (r = −0.07, *p* = 0.84). Figure 2 shows the scatter plot of right and left SHIMP gains across years of dance experience.

### 3.3. Anti-Compensatory Saccade Latency

**Figure 3:**
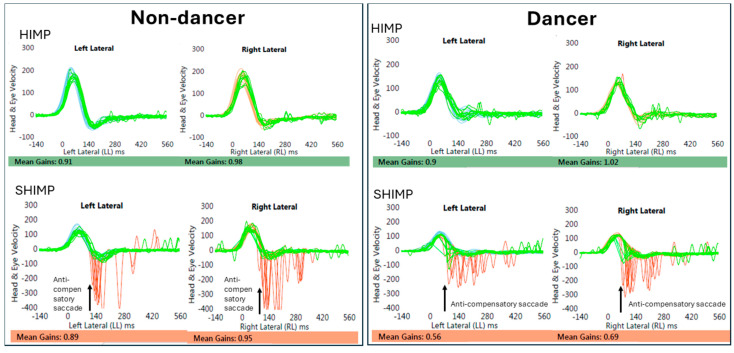
Eye (red) and head movement (green) traces during the HIMP and SHIMP for a non-dancer and a dancer (with 16 years of training). The HIMP shows similar VOR gains between the two groups. In the SHIMP (lower panel), the onset of anti-compensatory saccade latency (indicated by the arrow) is seen to be about 100 ms for the non-dancer, and about 75 ms for the dancer for a few runs.

Anti-compensatory saccades were observed in the SHIMP for both dancers and non-dancers. While there was not a statistically significant difference in SHIMP gain values between the groups, three dancers with over 15 years of training showed an earlier onset of anti-compensatory saccadic latency (see example in Figure 3). In these three dancers, for some of the head movements, the anti-compensatory saccades appear to begin (before 80 ms), while the head is still in motion. For other participants, anti-compensatory saccades began only after head motion had stopped.

## 4. Discussion

In this study, all participants demonstrated VOR measures within normal limits, and the overall statistical model revealed no significant differences between dancers and non-dancers in HIMP or SHIMP gain. However, evidence of changes in the VOR was observed. A significant negative correlation was found between right-side SHIMP gain and years of dance training in dancers, while no such relationship was observed for left-side SHIMP gain or HIMP gain on either side. Additionally, three participants with over 15 years of dance training exhibited anti-compensatory saccade latencies of approximately 75 ms during SHIMP, where the anti-compensatory saccade occurred before the completion of the head movement.

### 4.1. The Effects of Dance Training on the HIMP and the SHIMP Gain

Although no significant group differences were found in HIMP or SHIMP gains, dancers showed greater variability in SHIMP VOR gains. When analyzed as a continuous variable, years of dance experience correlated with a decrease in SHIMP VOR gain, particularly for right-sided SHIMP, aligning with most dancers’ preferred turning direction. While our study found reduced SHIMP gain with increased dance experience, we did not observe a significant group effect, which contrasts with Maheu et al. (2019) [18]. One of the reasons for this discrepancy may be due to differences in VOR calculation methods—we used the area ratio gain, which measures eye movement throughout the entire head movement, whereas Maheu et al. (2019) [18] used instantaneous gain. Notably, Maheu et al. (2019) [18] found a significant group effect only at 60 ms, with no effect at 40 or 80 ms. Since VOR suppression during high-frequency head movement typically begins after 80–90 ms [21], using an area ratio gain becomes more meaningful as it traces the eye movement for the entire duration of head movement and not just at instantaneous time points (40, 60, and 80 ms).

Additionally, dancers in the Maheu et al. (2019) [18] study trained actively, averaging 16 h per week, and rated themselves at an advanced or expert level. In contrast, the dancers in this study were full-time college students with varied dance backgrounds who did not train as intensively as the dancers in Maheu et al. (2019) [18]. It is currently unknown how much training is required for VOR changes among dancers to begin. The current findings suggest changes in VOR suppression even among less active, non-professional dancers. These results substantiate the need for further research on VOR and VOR suppression in a larger sample with diverse levels of dance training to determine the amount of training required to influence VOR gain changes.

### 4.2. Anti-Compensatory Catch up Saccade

During the SHIMP, all participants in both groups demonstrated an anti-compensatory catch-up saccade (a reflexive eye movement back to the head-fixed target). In the three participants with over 15 years of dance experience, this anti-compensatory saccade began before the head movement was fully completed. Typically, the suppression of VOR begins at 80–90 ms for high-frequency passive head movement [21]. The earlier saccadic onset (about 75 ms) for some trials, among highly trained dancers, suggests an enhanced vestibulo-cerebellar circuitry, enabling the earlier initiation of the suppression of VOR during high-frequency head movements. Recently, Moin-Darbari et al. (2023) [19] reported an earlier onset of eye movements relative to head movement among long-term trained dancers during high-frequency HIMP VOR measurements. They reported a slightly higher VOR gain in trained dancers, attributing this to a phase lead in eye movement, with an average phase lead of up to 10 ms. The authors hypothesized that dancers may have stronger anticipatory control resulting in an early onset of eye movements during passive head impulses. We speculate that the modified eye–head timing during high-velocity movements, as described by Moin-Darbari et al. (2023) [19], may also be contributing to the earlier onset of VOR suppression, leading to earlier anti-compensatory saccade latency in highly trained dancers.

### 4.3. Asymmetry Between the Right and the Left VOR Gain

An additional noteworthy finding emerged in this study, though its relevance to the effects of dance training on VOR gain remains unclear. A significant difference was observed between left and right HIMP gains, with the right side exhibiting slightly higher gain than the left. In this study, the monocular camera was mounted on the right eye, which may have contributed to the relatively larger VOR gain recorded on the right. Similar findings have been reported in previous studies; for instance, McGarvie et al. (2015) found a slightly higher VOR gain on the right side (where the monocular camera was mounted) among 91 participants across age groups [22]. This was attributed to the increased demand on the VOR when the head moves towards the eye on which the camera is mounted. The authors explained that as the head rotates to the right, the right eye must rotate slightly more within the skull than the left eye to maintain fixation on an Earth-fixed target. Conversely, during leftward head movements, the right eye would need to rotate less than the left eye; however, since measurements are taken only from the right eye, the recorded VOR gain is relatively lower. Such asymmetry between the right and left sides using monocular camera (CNC Engineering, Seattle, WA, US [23]; Otometrics Impulse, Schaumberg, IL, US [24]; GN Otometrics, Denmark [25]) has been consistently reported across studies [23,24,25].

## 5. Implications

The earlier saccadic onset and reduced SHIMP VOR gain observed in dancers suggest a possible conscious modulation of the VOR network as an effect of dance training. Dancers demonstrate an extraordinary ability to perform multiple pirouettes with minimal or no sensation of vertigo, which has been linked to changes in cortico-vestibular-perceptual networks [11]. However, the same authors also reported a dissociation between VOR and activation of the vestibular perceptual network, noting that dance training had no effect on VOR when assessed using the STEP velocity test [11]. In the current study, we did not observe a significant difference in HIMP gain between dancers and non-dancers, but SHIMP gain decreased with increasing dance experience. A similar pattern of no effect on HIMP, but a significant effect on SHIMP, has been observed in dancers [18] and trained athletes [26] using vHIT.

The lack of change in VOR for HIMP measurements, alongside reduced VOR gain during SHIMP, suggests that dancers may have superior voluntary eye control depending on the task assigned. For HIMP, dancers were instructed to gaze at an Earth-fixed target, while for SHIMP, they were instructed to gaze at a head-fixed target. This task-specific control may explain the lack of effect on HIMP and the reduced gain on SHIMP. It is well established that dance enhances cognitive control [27], which is associated with enhanced sensory-driven motoric movements [14], potentially helping dancers better predict and manage eye movements during these tasks. Additionally, the ability to control eye movements is supported by findings from Schärli et al. (2024), where the voluntary use of spotting during 14 active and passive turns reduced dancers’ perception of dizziness, while the dancers felt dizzy when not using the technique [12]. Overall, we suggest that while dancers’ VOR may remain unaffected in HIMP tests, their ability to voluntarily control eye movements at high head velocities (similar to their training with spotting techniques) may be linked to the reduced SHIMP gain observed in our study.

## 6. Limitations and Future Directions

The current study has several limitations. First, the sample size was small, with only 18 participants. However, despite this limitation, the study provides preliminary evidence of reorganization changes in the VOR suppression, suggesting potential avenues for further research on the vestibular system in the context of dance training. While this study focused on VOR and its suppression at high-frequency natural stimulation, VOR operates across a range of frequencies, and it would be valuable to explore the effects of dance training across this spectrum. Furthermore, since VOR responses are also elicited in vertical planes of movement, examining these planes could provide a more comprehensive understanding. Future research could also address whether differences in SHIMP VOR gain apply similarly to male dancers, offering insights into potential gender-based variations. Additionally, different forms of dance training may affect the VOR in distinct ways. In the current study, the dancers were trained in various dance styles. It may be valuable to study a larger, more homogeneous group of dancers who consider themselves advanced in pirouettes or fouetté turns, as these dance forms may have a stronger impact on VOR. Finally, a longitudinal research design could offer a more effective understanding of changes in VOR resulting from dance training. Future studies examining quantified latency-based analyses of the SHIMP anti-compensatory saccade could offer deeper insights into VOR reorganization.

## 7. Conclusions

The current study found no difference in VOR gain between dancers and non-dancers. However, increased dance experience was negatively correlated with SHIMP gain. The study provides preliminary evidence suggesting that, while dancers have an intact and healthy VOR similar to non-dancers, they may also possess enhanced voluntary control over these reflexes, allowing them to suppress the VOR more effectively than non-dancers. As a preliminary study, future research with a larger sample size and a longitudinal design is needed to improve generalizability. Nonetheless, the outcomes of this study have opened opportunities to explore the potential benefits of dance-related brainstem-mediated vestibular reorganization.

## Data Availability

As per the Institutional Review Board (IRB) at James Madison University, at which the study was carried out, the data can only be shared with individuals approved by the Institutional Review Board.
